# Very High‐Power Short‐Duration Ablation for Atrial Fibrillation in Adults With Congenital Heart Disease

**DOI:** 10.1111/jce.16567

**Published:** 2025-01-24

**Authors:** Sarah Lengauer, Nico Erhard, Miruna A. Popa, Marta Telishevska, Hannah Krafft, Fabian Bahlke, Florian Englert, Felix Bourier, Tilko Reents, Isabel Deisenhofer, Gabriele Hessling

**Affiliations:** ^1^ Department of Electrophysiology, German Heart Center Munich TUM University Hospital Munich Bavaria Germany

**Keywords:** adults with congenital heart disease, long‐term outcome, paroxysmal and persistent atrial fibrillation, safety, very high‐power short duration ablation

## Abstract

**Introduction:**

Data regarding safety and long‐term outcome of very high‐power‐short duration (vHPSD) ablation in adult congenital heart disease (ACHD) patients with paroxysmal or persistent atrial fibrillation (AF) are lacking.

**Methods:**

Retrospective observational single‐center study. The data of 66 consecutive ACHD patients (mean age 60 ± 12.8 years, 46% male) with mild (69.7%), moderate (22.7%), or complex (7.6%) congenital heart disease (CHD) who underwent ablation for paroxysmal (40.9%) or persistent AF (59.1%) were analyzed. Circumferential PVI was performed in all patients and additional substrate ablation in 79,4% of persistent AF patients using irrigated RF energy with vHPSD settings of 70 W/5–7 s or 60 W/7–10 s.

**Results:**

Mean procedure time was 123.6 ± 42 min with a mean RF time of 18.19 ± 10 min. No technique related adverse events occurred. Vascular access complications were detected in seven patients (10.6%) requiring intervention in four patients (6%). A median follow‐up time of 491 days (IQR: 194–1054 days). Freedom from any atrial arrhythmia off antiarrhythmic drugs (AAD) at 1 year was present in 58% of patients (77.8% with paroxysmal AF, 43.6% with persistent AF).

**Conclusion:**

vHPSD for ablation of paroxysmal or persistent AF in ACHD patients is safe and effective. Regardless of CHD complexity, no vHPSD ablation modality related complications occurred. Long‐term outcome for paroxysmal AF after one ablation was excellent whereas results for persistent AF were limited.

## 1 Introduction

As therapeutic options in patients with congenital heart disease (CHD) have improved over the last decades [1], many of these patients encounter arrhythmias during adulthood [2]. In particular, atrial arrhythmias are a leading cause of morbidity and hospitalization in adult congenital heart disease (ACHD) patients [3, 4]. Atrial fibrillation (AF) has already surpassed intra‐atrial reentrant tachycardia (IART) as leading atrial arrhythmia in ACHD patients older than 50 years [4]. Catheter ablation is the therapy of choice, especially after failed attempts of rhythm control by antiarrhythmic drugs (AAD) [5].

Pulmonary vein isolation (PVI) using different radiofrequency (RF) energy techniques is a cornerstone of AF ablation and considered safe and effective [6]. Using RF energy for ablation, resistive heating causes local temperature rise to > 50°C leading to nonreversible myocardial tissue injury whereas conductive heating passively extends to deeper tissue layers causing potential reversible tissue damage/edema caused by local temperature rise < 50°C [7, 8]. A concern using RF energy is to afflict extra‐cardiac structures as the esophagus or phrenic nerve by uncontrolled lesion growth due to peripheral tissue heating. Data showing a beneficial shift from conductive to resistive heating by using very high power and short duration (vHPSD) RF applications have been published [9, 10]. Clinical data using vHPSD in patients without CHD showed improved long‐term efficacy [11]. As data using vHPSD in ACHD patients undergoing AF ablation are lacking, we analyzed the safety and efficacy of this new technique.

## 2 Methods

### 2.1 Study Population

The data of 66 ACHD patients (mean age 60 ± 12.8 years, male [46%]; mean CHA_2_DS_2_VASC Score 2.3 ± 1.4) who underwent catheter ablation using vHPSD for paroxysmal (*n* = 27/66; 40.9%) or persistent AF (*n* = 39/66; 59.1%) between May 2018 and February 2023 at our center were retrospectively analyzed. Patients with additional IART were not included in the study.

The study was approved by the local ethics committee (approval 2020‐348_1‐S‐NP).

Patient baseline and procedural characteristics were collected from the department's computerized database (Table 1). Congenital cardiac anomalies were classified as mild (*n* = 46; [69.7%]), moderate (*n* = 15; [22.7%]), or complex (*n* = 5 [7.6%]) CHD [12]. All 66 patients were on oral anticoagulation with apixaban (*n* = 33; 50%); edoxaban (*n* = 12; 18.2%), rivaroxaban (*n* = 8; 12.12%), dabigatran (*n* = 3; 4.5%), or phenprocoumon (*n* = 10; 15.1%). Congestive heart failure was present in 9 patients (13.6%), diabetes in 6 patients (9.1%), and 17 patients (25.7%) had a history of stroke (Table 1).

**Table 1:** Baseline characteristics.

	All patients (*n* = 66)	Mild congenital heart disease (*n* = 46)	Moderate congenital heart disease (*n* = 15)	Complex congenital heart disease (*n* = 5)
Age	60 ± 12.8	60 ± 12.77	62 ± 12.75	61 ± 13.43
Type of atrial fibrillation				
Paroxysmal	27 (40.9%)	22 (48.8%)	3 (20%)	1 (20%)
Persistent	39 (59.1%)	24 (52.1%)	11 (73.33%)	4 (80%)
CHA_2_DS_2_‐VASC Score	2.3 ± 1.4	2.37 ± 1.44	2.43 ± 1.49	2.49 ± 1.46
CHD classification		46 (69.7%)	15 (22.7%)	5 (7.6%)
Surgery (corrective or palliative)	24 (33.36%)	12 (50%)	9 (38.3%)	3 (12.5%)
Interventional treatment	17 (25.75%)	14 (82.35%)	2 (11.76%)	1 (5.88%)
No surgical or interventional treatment	27 (40.9%)	20 (74.07%)	5 (18.51%)	2 (7.4%)
Arterial Hypertension	28 (42.42%)	23	4	1
Diabetes	6 (9.09%)	5	1	0
Congestive heart failure	9 (13.63%)	6	2	1
BMI, kg/m^2^	27.53 ± 5.58	27.56 ± 5.59	27.16 ± 5.29	26.43 ± 4.36
History of stroke	17 (25.75%)	13	3	1

Abbreviations: BMI, body mass index; CHD, congenital heart disease.

### 2.2 Procedure and Ablation Protocol

An intra‐atrial thrombus was excluded before the procedure as described before Popa et al. [11]. Written informed consent was obtained. All procedures were performed under conscious sedation utilizing Propofol, Midazolam, and Fentanyl on uninterrupted oral anticoagulation (INR 2‐3 in patients on Vitamin K antagonists) (Table 2).

**Table 2:** Single and multiple procedure outcome following catheter ablation.

Arrhythmia type	Recurrence of AF or AT after one procedure	Freedom from AF or AT after one procedure
Paroxysmal AF (*n* = 21)	2	19
Persistent AF (*n* = 34)	20	14

Arrhythmia type	Recurrence of AF or AT after one or two procedures	Freedom from AF or AT after one or two procedures
Paroxysmal AF (*n* = 21)	0	21
Persistent AF (*n* = 34)	14	20

After gaining unilateral femoral venous access, a steerable multipolar catheter was placed inside the coronary sinus. Fluoroscopy guided single transseptal puncture with double left atrial access with a steerable 11.7‐Fr sheath (AgilisTM; Abbott) was achieved. Heparin was administered after successful transseptal puncture to gain an activated clotting time > 300 s during the procedure (Table 3).

**Table 3:** Classification of congenital heart disease.

	All patients (*n* = 66)	Simple CHD (*n* = 46)	Moderate CHD (*n* = 15)	Severe CHD (*n* = 5)
Atrioventricular septal defect	3		3	
Secundum Type atrial septum defect	38	38		
Surgical closure		7		
Interventional closure		13		
Native		18		
Coarctation of the aorta	4		4	
Congenitally corrected transposition of the great arteries	2			2
Ebstein's anomaly	3		3	
Patent Ductus arteriosus	2	2		
Sinus venosus atrial defect, partial anomalous pulmonary venous drainage	3		3	
Subaortic stenosis	1		1	
Tetralogy of Fallot s/p Conduit repair	2			2
Tricuspid valve atresia Type 1b/s/p Fontan Op	1			1
Ventricular septal defect	6	6		
Ventricular septal defect with mitral disease	1		1	

Three‐dimensional (3D) electroanatomical high‐density mapping and reconstruction was performed using EnSite Precision or EnSite X (Abbott) or CARTO 3 (Biosense Webster). An electroanatomical voltage map was created in either sinus rhythm or AF. Annotation of CFAE signals were visualized [13]. Low voltage for abnormal atrial areas was defined as an amplitude of < 0.5 mV.

Antral circumferential PVI was performed using a point‐by‐point ablation technique either with a 4 mm irrigated‐tip catheter FlexAbility SE/Ampere RF generator (Abbott) or a 3.5 mm irrigated‐tip catheter Thermocool Smarttouch SF/SmartAblate RF generator (Biosense Webster). During PVI, a circumferential or multipolar mapping catheter was placed inside the ipsilateral PV. The endpoint of PVI was defined as the absence of any PV spike potential recorded on the Lasso catheter and proof of exit block. Persistent AF was treated with additional substrate modification using vHPSD guided by LA voltage maps with the intention of AF cycle length prolongation or termination [14]. If this was not achieved, an external cardioversion was performed to restore sinus rhythm. Additional substrate ablation in low voltage areas was performed at the operator's discretion aiming for the isolation of these areas.

### 2.3 Ablation and Power Settings

Ablation and power settings were used with regard to the respective system. Using the Flexibility SE catheter, 70 W/7 s (anterior wall) or 70 W/5 s (posterior wall) were applied, whereas 60 W/10 s (anterior wall) or 60 W/7 s (posterior wall) were delivered with using the Thermocool Smarttouch SF catheter [15]. Automatic temperature cut‐off was set at 42°C (FlexAbility SE) and 40°C (Thermocool Smarttouch SF). Real‐time automated display of RF applications was employed with the following settings: For EnSite (Abbott), the AutoMark module was used (tag size 4 mm/minimum time 2 s for vHPSD and for CARTO3 (Biosense Webster) the Visitag function was enabled (tag size 3 mm, location stability 3 mm for 3 s and minimum contact force 25% of time > 3 g). An interlesion distance of 5–6 mm was targeted. Specific power and ablation settings were used as described previously [16] and were adapted to the specific catheter tip design.

### 2.4 Follow‐Up

All patients received a proton pump inhibitor (pantoprazole 40 mg twice daily) for 4 weeks following ablation and continued oral anticoagulation. Complications occurring within 30 days after ablation were assessed using in‐hospital monitoring, routine follow‐up visits at 1 month, or any unscheduled visits in our outpatient clinic. Pericardial effusion was routinely ruled out by transthoracic echocardiography at the end of each procedure and on the following day. TIA or stroke were defined according to current guidelines [17, 18]. Vascular access complications were assessed using sonography and defined following the consensus report from the Bleeding Academic Research Consortiums [19]. A blanking period of 30 days was applied. Any kind of arrhythmia recurrence was assessed during 3‐, 6‐ and 12‐month follow‐up visits using 7‐day‐Holter ECGs.

### 2.5 Statistical Analysis

Continuous variables are presented as mean ± standard deviation or median. Categorical data are expressed as frequencies and percentages. Univariate comparisons were performed using the *t* test (continuous variables) and the *χ* ^2^ test. A *p* value of < 0.05 was considered statistically significant. Cumulative event rates were calculated using the Kaplan–Meier method. A log‐rank test was performed to compare event distribution between both groups. All analyses were performed using SPSS for Mac version 20.0 (SPSS Inc.).

## 3 Results

### 3.1 Procedural Outcomes

Successful PVI using vHPSD was performed in all 66 patients using the CARTO 3 (*n* = 22, 33.3%) or the EnSite (*n* = 44; 66.7%) system. Additional substrate modification was performed in 31 (47%) persistent AF patients. Mean skin‐to‐skin procedure time was 123.6 ± 42 min (PVI only 108.43 ± 28 min, PVI+ substrate modification 147.27 ± 45 min; *p* = 0.53). Mean RF time was 18.19 ± 10 min (PVI alone 11.55 ± 3.47, PVI + CFAE 25.32 ± 9.57 min; *p* < 0.001) Mean power was 55.98 ± 3.51 W with a mean temperature of 32.49 ± 3.2°C. Mean fluoroscopy time was 9.04 ± 5.39 min (PVI only 8.06 ± 5.48 min; PVI + CFAE 10.03 ± 5.16 min; *p* = 0.16).

### 3.2 Complications

No adverse events such as cardiac tamponade, pericardial effusion > 10 mm, transient ischemic attack/stroke, atrio‐esophageal fistula, cardiac arrest, or death occurred peri‐interventionally or within the first 30 days after ablation. Vascular access complications occurred in seven patients (10.6%) including hematoma > 5 cm (*n* = 1), arteriovenous (AV) fistula requiring surgical treatment (*n* = 2), AV fistulas not requiring intervention (*n* = 1), or pseudo aneurysm (*n* = 3; thrombin injection in *n* = 2). Overall, four patients (6%) needed interventional treatment of a vascular access complication.

### 3.3 Follow‐Up and Outcomes

**Figure 1:**
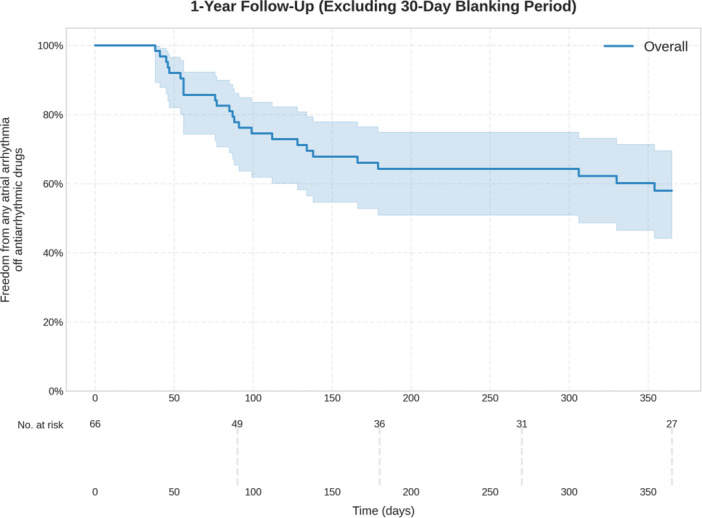
Single procedure outcome after vHPSD ablation (freedom from any atrial arrhythmia off antiarrhythmic drugs).

Follow‐up beyond the blanking period was available in 64/66 patients (96.9% with a median follow‐up duration of 491 days (IQR: 194–1054 days). At 1 year follow‐up, 58% of patients (95% CI: 44.2%–69.5%) were in stable sinus rhythm without AAD after a single ablation procedure (Figure 1).

Freedom from any atrial arrhythmia following a single ablation procedure after 1 year was 77.8% for patients with paroxysmal AF (95% CI: 57.1%–89.3%) as opposed to 43.6% for patients with persistent AF (95% CI: 29.3%–59.0%).

Following a second ablation, freedom from AF was 58.8% (20/34 patients) for persistent AF and 90% (19/21 patients) for paroxysmal AF patients.

## 4 Discussion

The present study presents first clinical data using vHPSD ablation in ACHD. It shows that the technique is feasible and safe in this patient population. Long‐term results are excellent for ACHD patients with paroxysmal AF after a single ablation procedure whereas long‐term outcome is limited in patients with persistent AF.

### 4.1 Feasibility and Safety of vHPSD Ablation

Ablation protocols using high power short duration ablation for AF have gained increasing interest within the last years [20, 21, 22]. High‐power delivery over a short period of time has shown to create more efficient lesions by a shift of increased restrictive heating versus decreased conductive heating in contrast to standard ablation protocols using 30–40 W irrigated RF energy [9, 10]. Increased safety and shorter procedure times have been reported [22]. A meta‐analysis [23] including 29 studies dealing with vHPSD/HPSD versus conventional power ablation in a non‐CHD population showed a shorter procedure time with vHPSD. Procedure and RF times in this study with ACHD patients were comparably low. This leads to a decreased usage of sedatives and X‐ray dosage/time as well which might be especially beneficial in ACHD patients where sedation and protracted procedure times present a challenge [24]. Recent studies [25, 26] have shown that vHPSD/HPSD is safe and effective with lower risk of esophageal injury compared with conventional RF energy delivery. Also in our study, no major technique‐related complications occurred regardless of CHD complexity.

The vascular access complication rate was quite high in our study. This might be mainly due to the fact that we did not routinely use ultrasound‐guided access before 2020. Additionally, all patients with mild hematoma or pain received a routine in‐hospital ultrasound of the groin vessels on the first day postablation, which might partially explain a higher overall detection rate. Similar findings were previously published by our group [27].

### 4.2 One Year Outcome of vHPSD Ablation

There are data indicating that in patients without CHD, the use of vHPSD leads to better long‐term results [21].

Acute and 1‐year success in our study was high (77,8%) paroxysmal AF patients. In contrast, a single center study including 57 ACHD patients with paroxysmal or persistent AF using a conventional irrigated RF ablation technique (maximum of 30 W) showed single procedure arrhythmia‐free survival rates of 63% at 1 year and 22% at 5 years [28]. Achieving a complete PVI is decisive for long‐term freedom from AF and vein reconnection is related to nontransmural lesions and tissue edema [29, 30]. Here vHPSD might offer a great advantage over conventional techniques.

Long‐term success in ACHD patients with persistent AF was limited in our cohort but improved with a second ablation. A reason for the limited outcome probably lies in both trigger and substrate perpetuating AF in ACHD patients. Triggers outside the pulmonary veins including the right atrium or superior vena cava might be of greater importance than in non‐CHD patients [31, 32, 33, 34]. Structural remodeling through atrial fibrosis in the right and left atrium from previous surgeries, atrial enlargement caused by residual septal defects, valvular disease, ventricular dysfunction, or other acquired conditions may play a role [35, 36, 37]. Chronic atrial pressure and/or volume overload favors triggered activity, resulting in focal atrial activity that triggers AF [38, 39]. Larger studies dealing with trigger and substrate modification in ACHD patients with persistent AF are required and may lead to more individualized ablation strategies in these patients.

### 4.3 Limitations

Patients with complex ACHD were underrepresented in our cohort. We presume that the relatively low number of ACHD patients with moderate and complex disease might be the result of excluding patients with additionally documented intra‐atrial‐reentrant tachycardia. As Labombarda [40] showed, the prevalence of IART increases with ACHD complexity. AF occurrence increases with patients age and was associated with fewer cardiac surgeries.

We did not include ablation settings other than the ones reported in the Methods section and did not apply a 90 W/4 s ablation setting. However, in concordance with previous studies, we would label power delivery > 50 W as “vHPSD” [41].

## 5 Conclusion

vHPSD for ablation of paroxysmal or persistent AF in ACHD patients is safe and effective. Regardless of CHD complexity, no vHPSD ablation modality related complications were observed. Long‐term outcome for paroxysmal AF after one ablation is excellent whereas results for persistent AF are limited. Larger multicenter trials are necessary especially for ACHD patients with persistent AF to define a more individualized ablation strategy for this patient population.

## Conflicts of Interest

The authors declare no conflicts of interest.

## Data Availability

The data that support the findings of this study are available on request from the corresponding author. The data are not publicly available due to privacy or ethical restrictions.
